# The Role of Growth Hormone in Depression: A Human Model

**DOI:** 10.3389/fnins.2021.661819

**Published:** 2021-06-24

**Authors:** Mubarak Algahtany, Shubham Sharma, Khalid Fahoum, Rowan Jing, Stanley Zhang, Kalman Kovacs, Fabio Rotondo, John Lee, Irene Vanek, Michael D. Cusimano

**Affiliations:** ^1^Division of Neurosurgery, Department of Surgery, College of Medicine, King Khalid University, Abha, Saudi Arabia; ^2^Keenan Research Centre for Biomedical Science, St. Michael’s Hospital, Toronto, ON, Canada; ^3^Faculty of Medicine, Queen’s University, Kingston, ON, Canada; ^4^Weil Cornell Medicine, New York, NY, United States; ^5^Indicator Research and Development Department, Canadian Institute for Health Information, Toronto, ON, Canada; ^6^Department of Laboratory Medicine and Pathobiology, St. Michael Hospital, University of Toronto, Toronto, ON, Canada; ^7^Division of Head and Neck Surgery, Department of Surgery, St. Michael Hospital, University of Toronto, Toronto, ON, Canada; ^8^Division of Neurosurgery, Department of Surgery, St. Michael Hospital, University of Toronto, Toronto, ON, Canada

**Keywords:** acromegaly, depression, growth hormone, human, transsphenoidal surgery, neuropeptides, brain

## Abstract

**Background:**

Although the relationship between acromegaly and depression has been ascribed to the effects of chronic disease, the role of growth hormone (GH), and insulin like growth factor-1 (IGF-1) is not clear.

**Objective:**

To determine whether related hormones levels in acromegalics are correlated with depressive symptoms and whether these symptoms are ameliorated following surgery.

**Materials and Methods:**

A prospective cohort study was conducted on patients diagnosed with acromegaly (*n* = 15) or non-functioning pituitary adenomas (NFPA; *n* = 20, as controls) and undergoing first-time surgery, who completed the Center for Epidemiological Studies Depression (CES-D) questionnaire both pre-surgery and post-surgery. The primary outcome was the patient’s severity of depression symptomatology using the CES-D score; GH, IGF-1 levels, and tumor characteristics were also measured.

**Results:**

Hormone levels (GH and IGF-1) and depression scores in acromegaly patients showed significant reductions following surgery (*p* < 0.05). The average change in CES-D score was 5.73 ± 2.58 (mean ± SE). A moderate correlation was found between GH levels and CES-D scores (*r* = 0.52, *p* < 0.01). The depressed affect subscale accounted for the most improvement in CES-D scores postoperatively and correlated most highly with GH levels. We did not find similar declines in the matched cohort of NFPA patients.

**Conclusion and Relevance:**

Surgical resection of the pituitary tumor in acromegaly patients leads to reduction in GH levels that is correlated with reduction in CES-D scores. The results suggest a role for GH in depression and provide a stronger foundation on which to build the hypothesis that GH impacts affect. The study also suggests that hormones should be factored into the matrix that entails the neuro-biological underpinnings of depressive disorders. Future work could explore the mechanisms involved, further brain and neuropeptide interactions, and, novel potential therapeutic targets in depressive and other mental health disorders.

## Introduction

The hallmark of acromegaly caused by a pituitary adenoma is excessive growth hormone (GH) secretion. Depression is common among acromegalics and contributes to their decreased quality of life (Geraedts et al., 2015; Sievers et al., 2009). In these patients, the related hormonal disturbance -above and beyond the physical changes induced by the disease- may play a significant role in the pathophysiology of depression. Although attention to the endocrine system’s role in the pathophysiology of depression is mounting, it has primarily focused on the hypothalamic-pituitary-adrenal axes (Juruena et al., 2018; Krogh et al., 2010). The role of growth hormone in the pathophysiology of depression, however, has not been sufficiently studied. This work aims to study the impact that surgery has on the depressive symptoms in patients with GH-secreting pituitary adenomas and relate it to GH and IGF-1 levels.

## Materials and Methods

### Study Design and Patient Sample

This was a prospective cohort study. The final sample consisted of 35 patients that included fifteen acromegaly patients and a set of twenty non-functioning pituitary adenoma (NFPA) patients (matched to the acromegaly group on age, sex, and level of education) who underwent endoscopic transsphenoidal surgery for the first time by the senior author between the year 2009 and 2016 (Table 1A; Fathalla et al., 2015; Cusimano et al., 2012). The NFPA patients were used to control for confounding factors such as the effects of mass effects and the impact of the knowledge of having a pituitary tumor, and the effects of surgery. In this way we intended to test whether excessive GH was important in the etiology of depression, as opposed to the effects of the pituitary tumor and its surgical treatment *per se*. The diagnosis of acromegaly was made according to the Endocrine Society’s criteria (Katznelson et al., 2014). All acromegaly patients had the characteristic neuroendocrine clinical presentation, confirmed by non-suppressible GH and elevated IGF-1 in the presence of a pituitary adenoma on high-resolution magnetic resonance imaging (MRI). The institutional ethics committee approved the study and informed written consent was obtained from each participant. For analyses, the patients were divided into the age groups of young (15–24), middle (25–64), and old (65+), similar to the categorization of depression data in the general Canadian population by Statistics Canada (Pearson et al., 2013).

**TABLE 1A:** Demographic information for acromegaly (*n* = 15) and NFPA (*n* = 20) patients.

		Acromegaly n (%)	NFPA n (%)	*p*-value
Sex	Male	6 (40)	11 (55)	0.38
	Female	9 (60)	9 (45)	
Level of education	6–12 years of schooling	4 (27)	3 (15)	0.73
	University/College BA/BSc	4 (27)	8 (40)	
	Graduate/Professional degree	3 (20)	5 (25)	
	No Response	4 (27)	4 (20)	
Age group	15–24	1 (7)	0 (0)	0.29
	25–64	13 (87)	16 (80)	
	65+	1 (7)	4 (20)	

*NFPA, non-functioning pituitary adenoma.*

All participants were requested to voluntarily complete a package of validated questionnaires, including the Center for Epidemiologic Studies Depression Scale (CES-D), before and after their surgery, as well as at each follow-up visit. Our initial acromegaly patients sample consisted of 66 unique patients who underwent surgery throughout the study period; however, some did not complete all the questionnaires, and because we wanted to eliminate potential effects of prior surgery or radiation, we excluded patients undergoing surgery for recurrent acromegaly. Fifteen of those remaining patients completed the questionnaires with complete data before and after surgery, allowing pairwise comparison of results.

The CES-D is a well-established, self-report screening test for depression in the general population (Radloff, 1977). A minimum CES-D score of 16 (out of 60) is classified as depression, where a higher CES-D score corresponds to more depressive symptomatology (Fathalla et al., 2014; Radloff, 1977). The CES-D scale can be divided into four subscales: depressed affect (D), positive affect (P), somatic (S), and interpersonal (I) (Mackinnon et al., 1998).

### Data Collection

We collected data on the patient’s age, sex, and level of education, as well as CES-D scores, anterior pituitary hormone levels, the largest tumor dimension on MRI, and presence of optic compression. We selected the CES-D questionnaire entries closest to the date of the surgery to standardize the data. The GH and IGF-1 levels were those collected at the time closest to the administration of the CES-D questionnaire, as well as those that corresponded to the date of the surgery for pituitary tumor resection.

### Analyses

Descriptive statistics were used to describe the patient population and paired t-test was used to compare the changes in the CES-D scores, GH levels, and IGF-1 levels for each patient (*n* = 15). Linear regression was used to explore the potential factors influencing changes in the CES-D score, including GH levels, IGF-1 levels, tumor size, and presence of optic chiasm compression. Pearson correlation was used to test the relationship between the CES-D and hormone levels. All analyses were performed using SAS 9.4 (SAS Institute, Inc., Cary, NC, United States). A *p*-value of less than 5% was considered statistically significant.

## Results

### Acromegaly Patient Characteristics and CES-D Scores

There was an even distribution regarding the sex and level of education among the acromegaly and NFPA patients; whereas, both patient groups were populated in the majority by the middle-aged subjects (Table 1A). The mean time between completing the pre-surgery questionnaire and surgery was 90 days, while the mean time between surgery and completing the post-surgery questionnaire was 114 days. The mean CES-D scores for males and females pre-surgery are statistically equivalent and decrease following surgery.

The differences in the CES-D score, GH levels, and IGF-1 levels pre-surgery vs. post-surgery were evaluated for each of the acromegaly patients (Table 1B). All three parameters showed significant reduction following surgery (*p* < 0.05) (Table 2). The CES-D scores showed a significant mean reduction of 5.73 points following surgery, reflecting an improvement in depression score. Meanwhile, the average unit-reduction in GH and IGF-1 levels were 15.82 and 573, respectively. Using Pearson’s correlation coefficient, we found a moderate correlation between GH levels and CES-D score (*r* = 0.52, *p* < 0.01) and a strong correlation between GH levels and IGF-1 levels (*r* = 0.69, *p* < 0.01), but a non-significant weak correlation between IGF-1 levels and CES-D score (*r* = 0.31, *p* > 0.1) (Table 3). The largest tumor dimension was significantly correlated to both GH and IGF-1 levels (*r* = 0.63, *p* < 0.05). Note that the correlations were computed using each measurement as a point, regardless of surgical status, hence the maximum sample size of *n* = 30 reported in Table 3, which corresponds to the 15 acromegaly patients with 2 measurements each, pre-surgery and post-surgery.

**TABLE 1B:** Summary statistics of measurements for acromegaly patients (*n* = 15).

	Pre mean (SD) [min, max]	Post mean (SD) [min, max]
CES-D Score	10.93 (9.66) [0, 30]	5.20 (5.52) [0, 21]
GH levels*	19.24 (15.45) [2.60, 49.90]	2.60 (6.97) [0.10, 26.70]
IGF-1 levels**	854.33 (253.64) [328, 1122]	320.23 (157.66) [43, 646]

There is some missing data in the hormone level recordings, so the sample sizes are: *n = 10 pre, *n* = 14 post; **n = 9 pre, *n* = 13 post. CES-D, Center for Epidemiologic Studies Depression Scale; GH, growth hormone; IGF-1, insulin-like growth factor-1; SD, standard deviation; min, minimum; max, maximum.

**TABLE 2:** Analysis of change scores (pre-post) in acromegaly patients.

	Change score mean (SE) [min, max]	95% CI	*p*-value
CES-D Score (*n* = 15)	5.73 (2.58) [−2, 29]	(0.21, 11.26)	0.043
GH levels (*n* = 10)	15.82 (4.66) [2.5, 49.8]	(5.29, 26.35)	0.008
IGF-1 levels (*n* = 9)	573 (78.63) [244, 883]	(387.1, 758.9)	<0.001

*CES-D, Center for Epidemiologic Studies Depression Scale; GH, growth hormone; IGF-1, insulin-like growth factor-1; SE, standard error; min, minimum; max, maximum.*

**TABLE 3:** Correlation between measurements for acromegaly patients.

	CES-D score total	GH level	IGF-1 level	Largest dimension	Optic compression status	CES-D subscale (Somatic)	CES-D subscale (Depressive)	CES-D subscale (Positive)	CES-D subscale (Inter-personal)
CES-D total	1.00 *n* = 30	0.52 (0.0093) *n* = 24	0.31 (0.158) *n* = 22	0.30 (0.181) *n* = 21	0.16 (0.431) *n* = 27	0.86 (< 0.0001) *n* = 30	0.90 (< 0.0001) *n* = 30	0.63 (0.0002) *n* = 30	0.64 (0.0001) *n* = 30
GH	0.52 (0.0093) *n* = 24	1.00 *n* = 24	0.69 (0.0005) *n* = 21	0.63 (0.0118) *n* = 15	−0.31 (0.165) *n* = 22	0.22 (0.293) *n* = 24	0.54 (0.006) *n* = 24	0.69 (0.0002) *n* = 24	0.24 (0.257) *n* = 24
IGF-1	0.31 (0.158) *n* = 22	0.69 (0.0005) *n* = 21	1.00 *n* = 21	0.63 (0.0123) *n* = 15	−0.16 (0.494) *n* = 20	0.23 (0.308) *n* = 22	0.29 (0.189) *n* = 22	0.26 (0.252) *n* = 22	0.34 (0.116) *n* = 22
Largest dimension	0.30 (0.181) *n* = 21	0.63 (0.0118) *n* = 15	0.63 (0.0123) *n* = 15	1.00 *n* = 21	−0.04 (0.870) *n* = 20	0.18 (0.428) *n* = 21	0.16 (0.484) *n* = 21	0.33 (0.141) *n* = 21	0.12 (0.607) *n* = 21
Optic compression status	0.16 (0.431) *n* = 27	−0.31 (0.165) *n* = 22	−0.16 (0.494) *n* = 20	−0.04 (0.870) *n* = 20	1.00 *n* = 27	0.31 (0.120) *n* = 27	0.09 (0.641) *n* = 27	−0.06 (0.768) *n* = 27	−0.01 (0.974) *n* = 27

*The values in this two-way table are presented as correlation coefficient (*p*-value). CES-D, Center for Epidemiologic Studies Depression Scale; GH, growth hormone; IGF-1, insulin-like growth factor-1.*

When analyzed by subscales, only the Depressed affect (D) and Somatic (S) subscales of the CES-D scores decreased following surgery, with mean decreases of 2.13 and 2.32, respectively (Table 4). Furthermore, among the tumor characteristics and hormone levels, only GH levels were significantly correlated with any of the subscales, namely Depressed affect (D) and Positive affect (P) (Table 3). Thus, it is evident that GH has a strong association with the (D) subscale, which significantly decreases following surgery, as GH levels drop, accounting for a decrease in the overall CES-D score.

**TABLE 4:** Analysis of change scores (pre-post) for CES-D subscales in acromegaly patients (*n* = 15).

	Change score mean (SE) [min, max]	95% CI	*p*-value
Depressed affect	2.07 (0.97) [0, 12]	(−0.01, 4.14)	0.05
Positive affect	1.27 (0.74) [−3, 7]	(−0.32, 2.85)	0.11
Somatic affect	1.87 (0.80) [−2, 10]	(0.14, 3.59)	0.04
Inter-personal affect	0.2 (0.17) [−1, 2]	(−0.17, 0.57)	0.27

*CES-D, Center for Epidemiologic Studies Depression Scale; SE, standard error; min, minimum; max, maximum.*

We ran a multiple linear regression to quantify the predictive value of these factors on depression, as indicated by the CES-D scores, but found no significant predictors for CES-D. However, the parameter estimate for GH was 0.66 (*p* = 0.15), which indicates a 0.66-unit increase in CES-D score for an increase in GH by 1 ng/ml. Although this predictor has a respectable *p*-value, it is not statistically significant, which may be explained by the limitation of a small sample size that prevents the functionality of the predictive model.

### Comparison With NFPA Controls

We explored whether the results observed were a consequence of either the presence of a pituitary tumor or the outcome of transsphenoidal surgery, rather than the GH excess specific to acromegaly, by comparing the results for acromegaly patients to NFPA patients matched on age, sex, and level of education. Mean pre-surgery CES-D scores were equivalent for both groups (Acromegaly = 10.93 vs. NFPA = 10.85; *p* > 0.9). However, mean post-surgery CES-D scores decreased for acromegaly patients but not for NFPA patients (Acromegaly = 5.20 vs. NFPA = 10.70; *p* = 0.26). While acromegaly patients had a significant mean difference of CES-D scores following surgery (mean change score = 5.73, *p* < 0.05), NFPA patients did not achieve that level of significance (mean change score = 0.15, *p* > 0.9). Furthermore, the comparison of change scores between acromegaly and NFPA patients was significant (Savage One-Way Analysis, *p* < 0.05) (Table 5). Moreover, when we categorized the CES-D as greater or less than 16 – the score indicative of clinical depression – 20% of acromegaly patients had a pre-surgery score greater than 16 and this declined to 6.7% post-surgery. The corresponding results for NFPA patients remained at 20% for both pre-surgery and post-surgery.

**TABLE 5:** Difference in mean CES-D scores (pre-post) comparison between acromegaly (*n* = 15) and NFPA (*n* = 20) patients.

	Change score mean (SD)	*p*-value (within group)	*p*-value (between group)
Acromegaly (*n* = 15)	5.73 (9.98)	0.04	0.02
NFPA (*n* = 20)	0.15 (9.14)	0.92	

*CES-D, Center for Epidemiologic Studies Depression Scale; NFPA, non-functioning pituitary adenoma; SD, standard deviation.*

The difference between NFPA and acromegaly patients was that acromegalics had pre-surgery high GH and IGF-1 levels and had smaller tumor sizes on average (Acromegaly = 1.84 cm vs. NFPA = 2.66 cm; *p* < 0.005). However, removing the larger tumors in NFPA patients did not affect their CES-D score, while the normalization of GH levels in acromegaly patients corresponded to a significant reduction in their CES-D scores.

## Discussion

In this study, we have taken the unique approach to assess the effects of surgery on depression ratings for a set of patients with growth hormone secreting tumors and compared the effects to a control group of patients with surgery for non-functioning pituitary tumors. We found that untreated acromegaly was associated with high CES-D scores, indicative of depression, and CES-D score improves following successful transsphenoidal surgery of GH-secreting pituitary adenoma. In paired analyses, GH levels, but not IGF-1 levels, were closely and significantly correlated with CES-D scores, and surgery had a profound effect on the decline in both absolute scores and the proportion of individuals classified as having clinical depression. We did not find similar declines in a matched cohort of NFPA patients. Neither the tumor size nor the optic compression status in acromegaly patients correlated with the CES-D score or any of its subscales. These findings suggest that GH may play a role in depressive symptomatology, more so than tumor size or the level of optic compression, and that acromegaly may be a human model for studying the role of the endocrine system in depression.

Previous studies have found that depression is more in acromegaly patients than other chronic somatic disorders and non-acromegalic control groups with similar comorbidities (Sievers et al., 2009; Crespo et al., 2015). These observations support our view that that depression in acromegaly is likely to be associated with levels of GH.

Our results indicate improvement in the CES-D score in the acromegaly patients but not in the NFPA control group and that the improvement was concordant with that of GH-level decrease, indicating some specificity in the role of GH in the etiology of depression in acromegaly patients. Depressed individuals have been found to have abnormal GH response to physical and psychological stress, marked by hypersecretion of GH compared to non-depressed individuals (Krogh et al., 2010). Further, GH supplementation’s positive effect on mood and memory of GH-deficient individuals has been shown (Arwert et al., 2005). Thus, it is conceivable that there is a strict balance in which both deficiency and excess of GH could lead to mood changes.

Growth hormone is a known neurotransmitter so our proposed hypothesis that GH has a role in the pathophysiology of depression in these patients is plausible biologically. GH has binding sites in the brain regions involved in depression, such as the hippocampus, thalamus, and hypothalamus, and it affects the functions of the central nervous system such as mood and cognition (Butler et al., 2019).

Our results show that the prevalence of preoperative depression in acromegaly patients was 20% and this dropped to 6.7% postoperatively, much closer to the 4.7% prevalence of depression in the Canadian population in any given 12-month period, as reported by Statistics Canada in 2012 (Pearson et al., 2013). On the contrary, the percentage of depressed NFPA patients remained constant at 20% for both pre-surgery and post-surgery. This further strengthens our presumption that acromegaly and NFPA groups are no different pre-surgery in terms of depressive symptomatology, but they are significantly different following surgery as acromegaly patients show a drastic decrease in their CES-D scores following a return to baseline in the GH and IGF-1 levels. Furthermore, this decrease in the CES-D scores is moderately correlated with a reduction of GH levels. The physiological effects of GH reduction after successful pituitary tumor surgery for acromegaly, such as on glucose tolerance, can be immediate but bony changes rarely reverse. We believe, therefore, that the positive effects of surgery on the depression that we have seen in our study subjects are related more to the improved physiological state than an improvement in physical effects since the mean time of post-operative assessment of CES-D score in our study is 4 months.

The current antidepressant drugs are based on the monoamine hypothesis of depression, have high complications, and low efficacy rates (Chávez-Castillo et al., 2019). This fact combined with the findings from our study calls for the need to study the neuroendocrine system as a possible treatment target. Some authors have suggested doing baseline neuroendocrine assessment to help predict the clinical response of depressed individuals to antidepressant drugs, given their high failure rate (Klimes-Dougan et al., 2018). This study proposes to look at depression as a complex neuroendocrine disorder and suggests GH as one endocrine target for studying depression with the potential to be a future therapeutic target. Future research should explore this inter-relationship in greater depth. This article builds on our knowledge of GH binding sites in the brain and directly links depressive symptoms with hormone levels rather than the mass effect of the tumor or the effects of surgery. It therefore highlights the intersection of hormones and neuropeptides in brain function and in mental health disorders.

## Limitations

Our study would have benefited from a larger sample size, which is generally a limiting factor for rare cases such as acromegaly. Our strict inclusion criteria could have introduced an element of selection bias into our results. However, we wanted patients with first surgery for acromegaly rather than introducing confounding factors with patients with recurrent acromegaly such as the effects of prior surgery and radiation. Voluntary completion of questionnaires may have contributed to selection bias. In order to mitigate the effects of confounding factors on outcomes, a novel aspect of our study is the inclusion of the NFPA control group. Despite the sample size we were able to show statistically significant results on the main outcome of interest. Increasing our sample size would have increased our statistical power and would have allowed us to run sub-group analyses.

Another limitation of the study is the non-standard timing of the GH-level collection. As GH levels cycle during the day, there may be variations depending on the time of measurement. However, these variations are small and the mean GH levels during the hours of interest fall within the range of 0–3 micrograms/liter (Surya et al., 2006). Consequently, the GH level variations do not have a substantial effect on our analysis but is a consideration worth noting.

Furthermore, as the mean time to collect CES-D questionnaires post-surgery was 114 days, there may be a confounding variable of sinonasal healing from surgery. This could have mitigated some of the improvement in CES-D scores depending on when the questionnaires were collected, however, the surgical technique was identical in our control group of NFPA patients, and no improvement in CES-D scores was noted there.

Additionally, some of the GH, IGF-1, tumor dimension, and optic compression data points were not available in the patient records for all patients at all time points, leading to some missing values as seen in the decreased sample sizes in Table 3. However, as the nature of the data is missing completely at random (MCAR), we can proceed without imputations. We can safely assume MCAR as these lab tests should have been performed on all acromegaly patients, regardless of their characteristics, physical, or hormonal. Therefore, any missing information can be assumed to not have been related to patient-level characteristics, but rather a result of inconsistent data entry. Any analysis that involves the variables with missing data simply does so over a smaller sample size than the one originally reported. These *n* values have been reported in the tables as necessary.

Finally, we understand and acknowledge that correlation does not imply causation, hence a larger sample size would also allow us to perform a multivariate analysis to explore the contribution of multiple factors to our outcomes. Future work should focus on corroborating our findings in larger multicenter studies and from diverse cultures that may approach depression differently or may have a more interconnected or disjointed support network.

## Conclusion

Transsphenoidal surgery to correct aberrant GH levels in patients with acromegaly leads to lower CES-D scores and a decreased percentage of clinical depression in this population. These CES-D scores are correlated with GH levels, with the decrease in depression corresponding to a decrease in GH levels. Our study supports the premise that GH plays a role in human depression, and highlights the fact that acromegaly may provide one human model to study depression. Ultimately, the findings clearly provide a stronger foundation on which to build the hypothesis that GH impacts affect and also that hormones should be factored into the matrix that entails the neuro-biological underpinnings of depressive disorders. Future work could explore the mechanisms involved, further brain and neuropeptide interactions, and novel potential therapeutic targets in depressive and other mental health disorders.

## Data Availability Statement

The raw data supporting the conclusions of this article will be made available by the authors, without undue reservation.

## Ethics Statement

The studies involving human participants were reviewed and approved by St. Michael’s Hospital, Toronto, ON, Canada. Written informed consent to participate in this study was provided by the participants’ legal guardian/next of kin.

## Author Contributions

All authors listed have made a substantial, direct and intellectual contribution to the work, and approved it for publication.

## Conflict of Interest

The authors declare that the research was conducted in the absence of any commercial or financial relationships that could be construed as a potential conflict of interest.
